# Influence of Oxygen Plasma on the Growth and Stability of Epitaxial NiCo_2_O_4_ Ultrathin Films on Various Substrates

**DOI:** 10.3390/ma15196911

**Published:** 2022-10-05

**Authors:** Kevin Ruwisch, Andreas Alexander, Tobias Pollenske, Karsten Küpper, Joachim Wollschläger

**Affiliations:** Department of Physics, Osnabrück University, 49076 Osnabrück, Germany

**Keywords:** nickel cobaltite, ultrathin films, RMBE, OPA-MBE, LEED, XPS, XRD, growth study, HAXPES

## Abstract

In this work, we investigated the influence of oxygen plasma on the growth of nickel cobaltite (NiCo_2_O_4_) thin films compared to growth in a molecular oxygen atmosphere. The films were grown on MgO(001), MgAl_2_O_4_(001) and SrTiO_3_(001) substrates by oxygen plasma (atmosphere of activated oxygen)-assisted and reactive molecular beam epitaxy (molecular oxygen atmosphere). Soft X-ray photoelectron spectroscopy showed that only the use of oxygen plasma led to a spectrum characteristic of (NiCo_2_O_4_). Low energy electron diffraction measurements were conducted to obtain information on the structure of the film surfaces. The results proved the formation of a spinel surface structure for films grown with oxygen plasma, while the formation of a rock salt structure was observed for growth with molecular oxygen. To determine the film thickness, X-ray reflectivity measurements were performed. If oxygen plasma were used to grow (NiCo_2_O_4_) films, this would result in lower film thicknesses compared to growth using molecular oxygen although the cation flux was kept constant during deposition. Additional X-ray diffraction experiments delivered structural information about the bulk structure of the film. All films had a rock salt bulk structure after exposure to ambient conditions. Angle-resolved hard X-ray photoelectron spectroscopy revealed a homogeneous depth distribution of cations of the grown film, but no typical (NiCo_2_O_4_) spectrum anymore. Thus, on the one hand, (NiCo_2_O_4_) films with a spinel structure prepared using activated oxygen were not stable under ambient conditions. The structure of these films was transformed into NiCo oxide with a rock salt structure. On the other hand, it was not possible to form (NiCo_2_O_4_) films using molecular oxygen. These films had a rock salt structure that was stable under ambient conditions.

## 1. Introduction

In the rising field of spin-based electronics (e.g., spintronics or spincaloritronics) transition metal ferrites and cobaltite are of the utmost importance due to their high Curie temperature and significant magnetic saturation moments. To fabricate high quality spintronic devices, it is essential that highly spin-polarized electron currents be generated. Here, nickel cobaltite (NiCo_2_O_4_, NCO) is a very promising candidate for this application. NCO has a ferrimagnetic ordering and predicted half-metallic ground state [1]. NCO crystallizes in an inverse spinel structure with a lattice constant in the range of 8.114 and 8.185 Å with a ferrimagnetic Curie temperature of 673 K [2,3,4,5]. Moreover, the tetrahedral sites could be occupied with Co^2+^ and Co^3+^ cations, while the octahedral sites are occupied with Ni^2+^, Ni^3+^ and Co^3+^ cation. A coupling between conductivity and cationic distribution (the degree of inversion defined as a fraction of Co^3+^ cations on tetrahedral sites) dependent on synthesis conditions has been reported [4], making p-type conducting NCO a promising candidate for future spintronic applications [4,6,7,8,9]. Further potential applications for NCO are, for example, infrared transparent electrodes [10,11] or supercapacitors [12,13] for energy storage [14,15,16].

In most studies, thin NCO films are deposited via sol-gel and thermal decomposition [17,18,19], pulsed laser deposition [4,10,20] or sputtering [21]. In this work, we used reactive molecular beam epitaxy (RMBE) and oxygen plasma-assisted molecular beam epitaxy (OPA-MBE) to prepare ultrathin NCO films. During the RMBE process, we used molecular oxygen while oxygen radicals were used during the OPA-MBE growth. In the following, we abbreviate RMBE prepared films with O_2_ and OPA-MBE prepared films with O*. Most commonly, NCO films are deposited on MgAl_2_O_4_(001) (*a*_MAO_ = 8.083Å, MAO) due to its small lattice mismatch of only 0.4–1.2% and no expected antiphase boundaries (APB) due to growth of spinel on spinel structures. In this work, we also used MgO(001) and (*a*_MgO_ = 4.2117 Å) SrTiO_3_(001) (*a*_STO_ = 3.905 Å) substrates to investigate the influence of the crystal structure of the substrates (rock salt and perovskite, respectively) on the growth of NCO thin films. For these substrates, the lattice mismatch was −3.7 to −2.87% and 3.9 to 4.8%, respectively. Compared to previous studies that prepared NCO, we used both RMBE and OPA–MBE as a new fabrication pathway. Both MBE techniques are used to grow epitaxial films, which in turn are thermodynamically most stable, since the energy of the adatoms on the surface is much lower than in other processes, such as PLD or sputter deposition [22,23,24]. Since a plasma source (OPA–MBE) is highly expensive, it would be of the highest importance to produce NCO samples without such an additional source. Directly after deposition, the chemical composition in the near-surface region and the surface structure of the films were determined using in situ soft X-ray photoelectron spectroscopy (soft-XPS) and low energy electron diffraction (LEED), respectively. After transport under ambient conditions, ex situ X-ray reflectivity (XRR) and X-ray diffraction (XRD) measurements were performed to analyze the structural bulk properties. To analyze chemical properties not only in the near-surface region but also for the bulk properties of the films, hard X-ray photoelectron spectroscopy (HAXPES) experiments were also performed.

## 2. Materials and Methods

Ultrathin NCO layers were prepared on MgO(001), MgAlO_2_(001) and SrTiO_3_(001) single crystalline substrates by using reactive molecular beam epitaxy (RMBE) and oxygen plasma-assisted molecular beam epitaxy (OPA-MBE) in an ultrahigh vacuum system with a base pressure of 10^−8^ mbar in the deposition chamber. We prepared the films using molecular oxygen (O_2_) and oxygen radicals O* by means of a SPECS plasma source. For cleaning purposes, the substrates were annealed at 400 °C in an oxygen atmosphere of 1 × 10^−4^ mbar for 1 h. Subsequently, XPS and LEED measurements were performed to monitor the chemical cleanliness and crystallinity of the surface. To grow the NCO thin films, two evaporators with pure nickel and cobalt rods were used to deposit the material on the substrates at a temperature of 250 °C in an oxygen atmosphere of 5 × 10^−6^ for both deposition types (RMBE, OPA-MBE). After film deposition, stoichiometry and surface structure were controlled by soft-XPS (Mg K_α_, *hv* = 1254 eV) using a SPECS Phoibos HSA 150 hemispherical analyzer and a LEED.After transport under ambient conditions, XRR and XRD measurements were performed at beamline P08 of Deutsches Elektronensynchrotron (DESY) [25]. For these measurements, a photon energy of 18 keV and a six-circle diffractometer with a two-dimensional PILATUS 100k detector was used. Additional HAXPES experiments were also performed at DESY at beamline P22 [26] using a SPECS Phoibos 225 HV hemispherical analyzer with a delay line detector and a wide angle lens. With this setup, the chemical composition of the films was investigated throughout the whole film, due to a high probing depth based on the energy of *hv* = 6000 eV. The information depth $D_{I}^{95}$ from where 95 of the detected photoelectrons were emitted could be calculated using the equation

(1) $$D_{I}^{95}\approx 3\lambda \cos\phi ,$$

where λ is the inelastic mean free path (IMFP), and ϕ is the off-normal emission angle. With the Tanuma, Powell and Penn algorithm formula we estimated the IMFP for various atomic orbitals [27]. The maximum information depth was given for an angle of Φ = 0° and is 21.5 nm (Co 2p) and 21.2 nm (Ni 2p), respectively. Compared to soft-XPS where the probing depth was only 3.1 nm for Ni 2p and 3.4 nm for Co 2p, and therefore sensitive to the surface, HAXPES delivered more bulk information.

## 3. Results and Discussion

### 3.1. LEED

**Figure 1:**
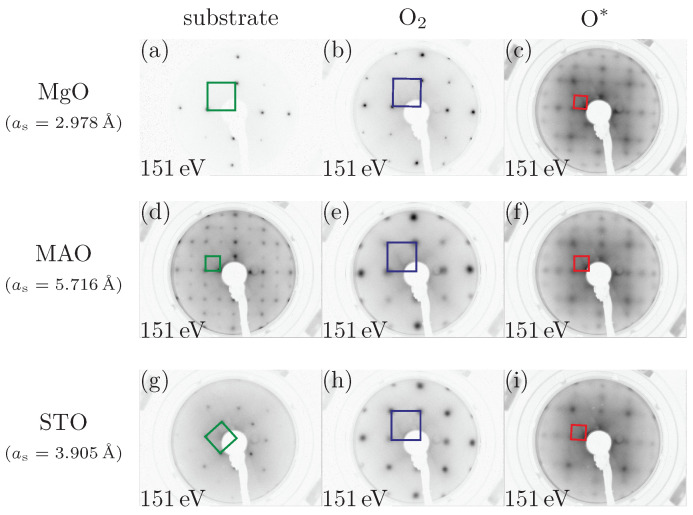
LEED patterns recorded at 151 eV for the substrates (first column: (**a**,**d**,**g**), the films prepared with O_2_ (second column: (**b**,**e**,**h**)) and the films prepared with O* (third column: (**c**,**f**,**i**)). $a_{s}$ are the surface lattice constants of the respective substrates. The squares represent reciprocal surface unit cells. The films prepared using O_2_ showed a (1×1) surface structure, which is too large to be a spinel structure, whereas the films prepared using O* showed a spinel structure.

LEED measurements were made directly after deposition of the NCO films. Figure 1 presents the LEED patterns recorded at an electron energy of 151 eV of the initially cleaned substrate surfaces, as well as the films with oxide films prepared using O_2_ or O*.

**Figure 2:**
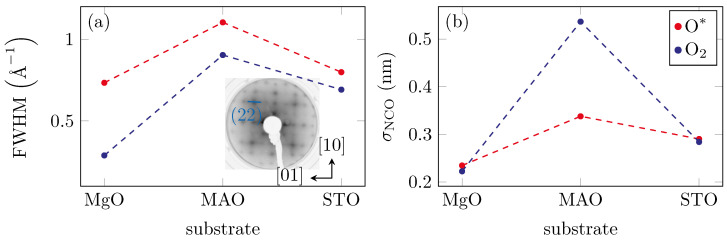
(**a**) FWHM of the (22) reflex with respect to the substrate (cf. inset). The FWHM was the highest for the MAO substrates. In general, the films with molecular oxygen exhibit a smaller FWHM than those with oxygen radicals. (**b**) RMS surface roughness $\sigma_{\mathrm{NCO}}$ of NCO. Here, also the surface roughness was the highest for the MAO substrate. In contrast to the FWHM, the surface roughness was higher for the films prepared with O_2_ than for the O* films.

The LEED patterns show sharp (1×1) diffraction spots for the substrates space.Looking at the films prepared with molecular oxygen, it was noticeable that the reciprocal surface unit cell was too large to be a spinel structure compared to the one in Figure 1d. Another indicator was that both the reciprocal unit cell of the MgO substrate (cf. Figure 1a) and that of the prepared film showed a comparable size (cf. Figure 1b). Therefore, the film with molecular oxygen could be either CoO with a surface constant of $a_{s}=3.012\;Å$ or NiO with a surface constant of $a_{s}=2.954\;Å$ or a mixture of both oxides. It was noticeable that sharp reflections were only discernible for the film on MgO, whereas the other two films showed much blurrier reflections (cf. Figure 2a), indicating a less ordered structure. A (1×1) structure was also evident in the films prepared in an oxygen plasma atmosphere. Moreover, these films exhibited a reciprocal surface unit cell similar to that of the MAO substrate that has spinel structure. Thus, the films prepared with oxygen plasma showed a spinel structure as expected for NCO films. For both kinds of preparation, an analysis of the full width at half maximum (FWHM) of the reflexes, as shown in Figure 2a, both quantifies and confirms the visual impression from the LEED patterns.

Obviously, the FWHM was much larger for the O* than for the O_2_ films. This indicated that more surface defects were created during the latter preparation. It could also be seen that the FWHM of the NCO film on the MAO substrate was generally higher than for the others. This seemed surprising since both the substrate and film have a spinel structure, and the lattice mismatch of the film and substrate was only 0.4 to 1.2%. Furthermore, we expected high quality films due to the matching of spinel on spinel without the formation of antiphase boundaries.

### 3.2. Soft-XPS

**Figure 3:**
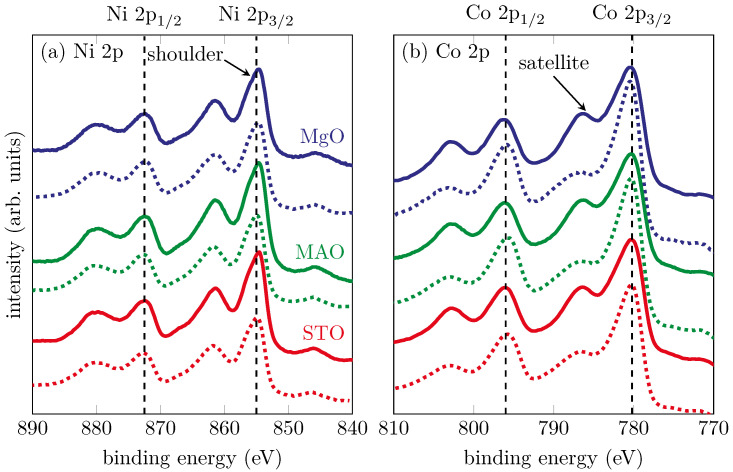
Soft-XP spectra of the (**a**) Ni 2p and (**b**) Co 2p core-levels. The solid lines represent the films prepared with O_2_ while the dashed lines represent the films prepared with O*. The vertical dashed lines represent the literature values for the respective main spin-orbit split peaks. The highlighted satellite is less distinguished for the films prepared with O*.

Soft-XPS measurements were made directly after deposition of the NCO films to investigate both stoichiometry and valence states of the cation species. Since soft-XPS was used with a low excitation energy and therefore a small information depth, this method delivered insight into the near-surface region. Figure 3 shows the core-level spectra of the Ni 2p and Co 2p orbitals. All spectra were calibrated according to the binding energy of the O 1s peak ($E_{B}=530$ eV).

All of the Ni 2p spectra showed a similar shape regardless of deposition in O_2_ or O*. All main peaks and all satellites appeared at the same binding energy. We found binding energies of 872.3±0.3 and 855.0±0.5 eV for Ni 2p${}_{1/2}$ and Ni 2p${}_{3/2}$, respectively. The distance between the satellites and the main spin-orbit split peaks is about 7 eV indicating the presence of Ni^2+^ cations [28]. The presence of the high binding energy shoulder of the Ni 2p${}_{3/2}$ peak was reported for the formation of NiO and therefore another indicator of the existence of Ni^2+^ cations [29].

For the spectra of the Co 2p region, we found a different behavior concerning the shape of the shake-up satellites. All of the main peaks and satellites appeared at the same binding energy, even for the films prepared with a plasma source O*. The positions of the main spin-orbit split peaks were at 796.0±0.4 eV and 780.1±0.3 eV for Co 2p${}_{1/2}$ and Co 2p${}_{3/2}$, respectively. Furthermore, the shakeup satellites are around a 6.5±0.5 eV higher binding energy than the correlated photoelectron peaks, indicating a Co^2+^ valence state [28,30,31]. However, here we found a difference in the shape of the spectra with respect to growth in O_2_ or O*. The high binding energy satellites of the Co 2p${}_{3/2}$ and Co 2p${}_{1/2}$ peaks were less distinguished for the films grown in O* than O_2_. As reported in the literature, this is evidence for the presence of Co^3+^ cations, as expected for the formation of NCO [18,32]. We could therefore state that using a plasma source changed the oxidation state of the Co valences because of exposure to O*.

For a quantitative analysis of the XPS data, the intensities $I_{\mathrm{Ni}}$ and $I_{\mathrm{Co}}$ of the entire Ni 2p and Co 2p spectra shown in Figure 3 were numerically integrated after subtracting a Shirley background. The relative Ni photoelectron yield $Y_{\mathrm{Ni}}$ was calculated by using the formula

(2) $$Y_{\mathrm{Ni}}=\frac{I_{\mathrm{Ni}}/\sigma_{\mathrm{Ni}}}{I_{\mathrm{Ni}}/\sigma_{\mathrm{Ni}}+I_{\mathrm{Co}}/\sigma_{\mathrm{Co}}},$$

after canceling the inelastic mean free path λ. The photoionization cross-sections $\sigma_{i}$ were taken from Trzhaskovskaya et al. [33] with respect to the excitation energy. For all films we found a yield corresponding to a stoichiometry of Co–Ni= 2:1 within uncertainties and as expected for the formation of NiCo_2_O_4_.

### 3.3. XRR

**Figure 4:**
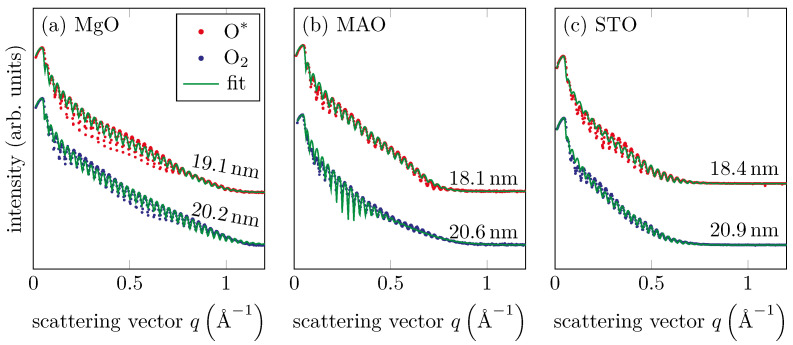
XRR measurements and fitted intensity curves of all films for (**a**) MgO substrate, (**b**) MAO substrate and (**c**) STO substrate. The fitted film thicknesses are shown on the respective curves. The films prepared with O* exhibited smaller film thicknesses compared to those prepared with molecular oxygen. All films prepared by the same technique showed comparable thicknesses.

XRR measurements were performed ex situ to determine film thickness as well as film roughness. Figure 4 shows the measured and calculated reflectivity curves after optimizing the structural parameters of the single-layer model using the Parratt algorithm [34] and the Névot–Croce roughness model [35]. The fitted curves were in good agreement with the experimental data. The resulting film thicknesses were depicted inside the figure. All samples grown in O* showed comparable film thickness within uncertainties. We also obtained this result for all samples grown in O_2_. Obviously, with the same evaporation parameters, the use of oxygen radicals for the growth of the films led to a smaller film thickness than with molecular oxygen. This may point to partial ion etching of the films grown in an O* atmosphere. Furthermore, the RMS roughnesses of the films were determined as presented in Figure 2b. We found values in the range between 0.2 nm and 0.6 nm and a similar behavior for the LEED FWHM, as significantly higher values occurred for the MAO substrate than for the others.

### 3.4. XRD

**Figure 5:**
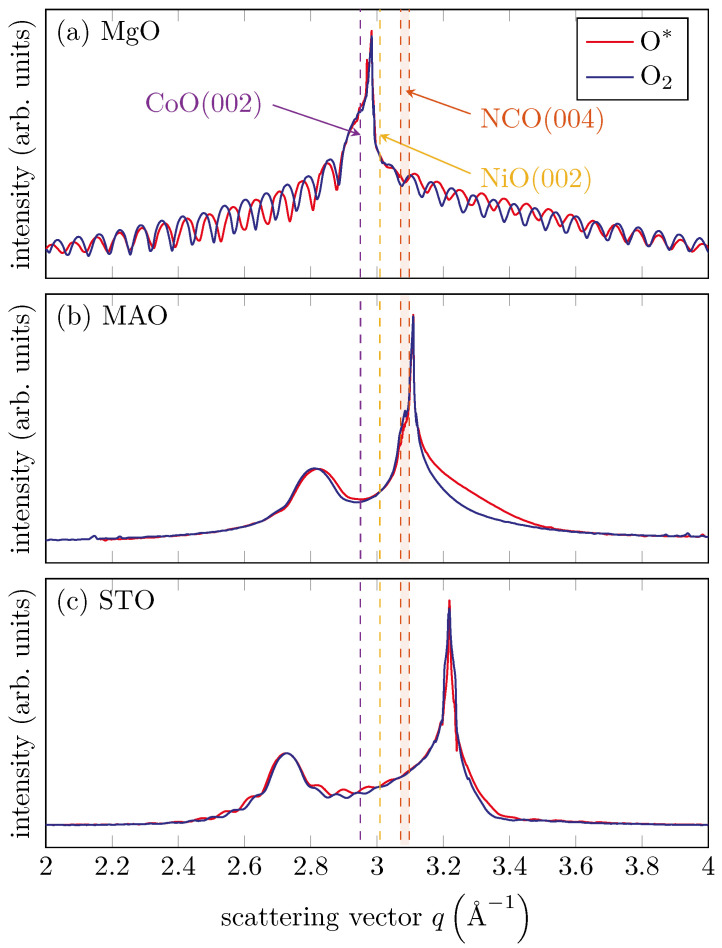
XRD measurements of the (00L) CTR across the (**a**) MgO(002), (**b**) MAO(004) and (**c**) STO(002) Bragg reflections. Dashed lines indicate the theoretical CoO(002) and NiO(002) Bragg peak positions for bulk material. Due to the possible lattice constants, a range of potential Bragg peak positions emerges for NCO(004). For (**a**) the film peak position agreed well with the theoretical value of CoO(002). In contrast, the film peak position for (**b**,**c**) show no agreement with the theoretical values.

XRD measurements in θ–2θ geometry were performed ex situ after the growth of the films to control their structural properties in detail. Figure 5 shows the results of the XRD measurements along the (00L) out-of-plane direction. The (00L) scans were measured along the MgO(002), MAO(004) and STO(002) Bragg reflections.

For the two films grown on the MgO(001) substrate using O_2_ or O* (cf. Figure 5a), we observed clear Laue fringes, indicating a high crystalline ordering and homogeneous thickness. The fringes were much less pronounced for films grown on the STO substrate (cf. Figure 5c) and nearly vanished for films grown on the MAO substrate (cf. Figure 5b). Therefore, we could state that the ordering of the oxide films depends heavily on the substrate. The ordering increases from films grown on MAO(001) to films grown on STO(001) while films deposited on MgO(001) had the highest ordering. This finding agreed well with our analysis of the FWHM of the LEED reflexes and of the surface roughness obtained from XRR. For a quantitative analysis, peak position and the FWHM of the oxide Bragg peaks were determined by fitting a Gaussian function. On the MAO substrate, we found next to the Bragg reflex of the substrate an additional diffraction peak of the oxide at a *q*-value of 2.821 Å^−1^ (O*) and 2.814 Å^−1^ (O_2_). The XRD data of the films on the STO substrate provided *q*-values of 2.726 Å^−1^ (O*) and 2.727 Å^−1^ (O_2_). For the films grown on MgO(001), we could only roughly estimate the film position to *q* = 2.948 Å^−1^ (O*) and 2.943 Å^−1^ (O_2_) from the Laue fringes since the Bragg peaks of the film and substrate strongly overlapped.

In addition, the dashed lines in Figure 5 showed the expected position for bulk CoO(002) and NiO(002). For NCO(004), we provide a range for the expected position due to the different possible lattice constants. Obviously, we obtained the best agreement for CoO films grown on MgO(001), but the Bragg positions of the films deposited on MAO(001) and STO(001) were shifted to lower values, indicating higher atomic layer spacings. This may have been attributed to an elastically distorted oxide film-to-lattice matching of film and substrate at the interface (pseudomorphic growth).

**Figure 6:**
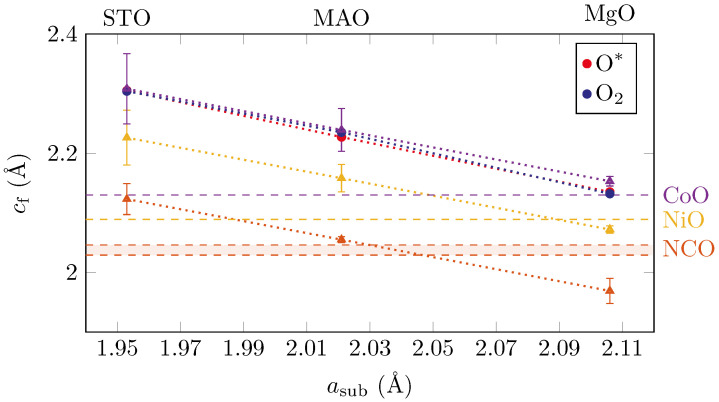
Vertical layer distance $c_{f}$ with respect to the lateral layer distance $a_{\mathrm{sub}}$ of the used substrates determined by the oxide film peak position. Furthermore, the theoretical vertical layer distances for bulk CoO, NiO and NCO are displayed with dashed horizontal lines along with the expected vertical layer distances for pseudomorphic growth on the different substrates (triangles).

Figure 6 shows the vertical layer distances $c_{f}$ obtained for the oxide films on the different substrates with respect to their lateral layer distances. The vertical layer distance $c_{f}$ of the films increased with decreasing lateral layer distances $a_{\mathrm{sub}}$ of the used substrates, as was expected for elastic distortion of the films.

The elastic vertical distortion $\Delta c_{f}$ of the film is related [36] to its lateral distortion $\Delta a_{f}$ via

(3) $$\frac{\Delta c_{f}}{c_{f,0}}=\frac{-2\nu }{1-\nu }\frac{\Delta a_{f}}{a_{f,0}}.$$

Here, $c_{f,0}$ and $a_{f,0}$ denote the bulk values of the vertical and lateral atomic layer spacing of the oxide film, and ν denotes the Poisson ratio. Assuming $\nu =\frac{1}{3}$ and pseudomorphic growth on the different substrates, the calculated vertical layer distances for the NCO, NiO and CoO films are presented in Figure 6 using triangles. Uncertainties of the calculated $c_{f}$ marked in Figure 6 are due to assumed uncertainties of the Poisson ratio $\Delta \nu =\pm \frac{1}{15}$.

The best agreement to the measured data was obtained for CoO films. Therefore, it could be speculated that the spinel structure of the NCO film grown in O* was transformed into a mixed CoNi oxide with a rock salt structure, as discussed in detail below.

The FWHM of the film Bragg peak was used to estimate the vertical crystallite size $D_{\mathrm{cryst}}^{\mathrm{vert}}$ using the Scherrer equation [37]. Crystallite sizes of 64.9 Å (O*) and 67.3 Å (O_2_) can be found for the films grown on the MAO substrate, which led to ∼33 of the calculated film thicknesses. After evaluating the FWHM of the film Bragg peaks on the STO substrate, the values for $D_{\mathrm{cryst}}^{\mathrm{vert}}$ of 77.4 Å (O*) and 84.1 Å (O_2_) and around 40% of the respective film thicknesses were. On the MgO substrate we estimated the vertical crystallite size using the distance between the Laue fringes and found 97.6 Å (O*) and 105.0 Å (O_2_), which was nearly 50% of the film thickness. Thus, the vertical crystallite size increased for growth on MAO(001) to STO(001) and was best for the growth on MgO(001). This result agreed well with the analysis of the surface roughness obtained by XRR as well as the analysis of the FWHM of the LEED reflexes.

**Figure 7:**
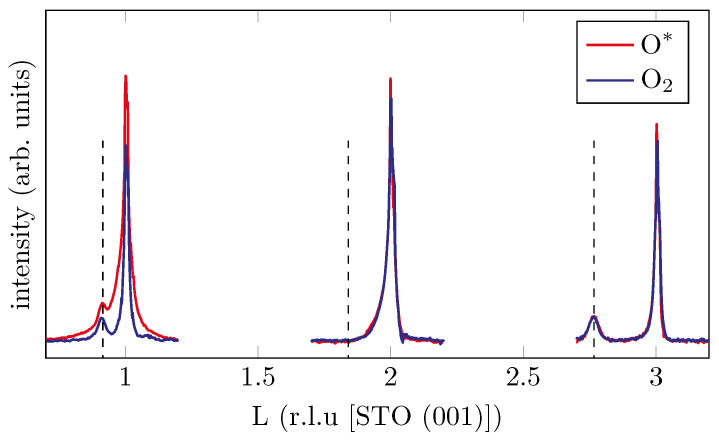
XRD measurements along (11L) CTR across the STO(11L) Bragg reflection. The dashed lines represent the peak positions of the oxide film. Bragg peaks can be identified close to the STO(111) and STO(113) reflex but not the STO(112). Thus, the oxide films have rock salt structure and do not show spinel structure.

Furthermore, XRD measurements were performed along the (11L) rod of each substrate to get information about the spinel structure of the oxide films. As an example, results for the STO substrate are shown in Figure 7.

On the STO substrate, we identified Bragg peaks close to the STO(111) and STO(113) reflex. However, there was no Bragg peak close to STO(112), indicating the absence of a spinel structure, but this finding can be explained by the formation of oxide films with a rock salt structure [38].

### 3.5. HAXPES

**Figure 8:**
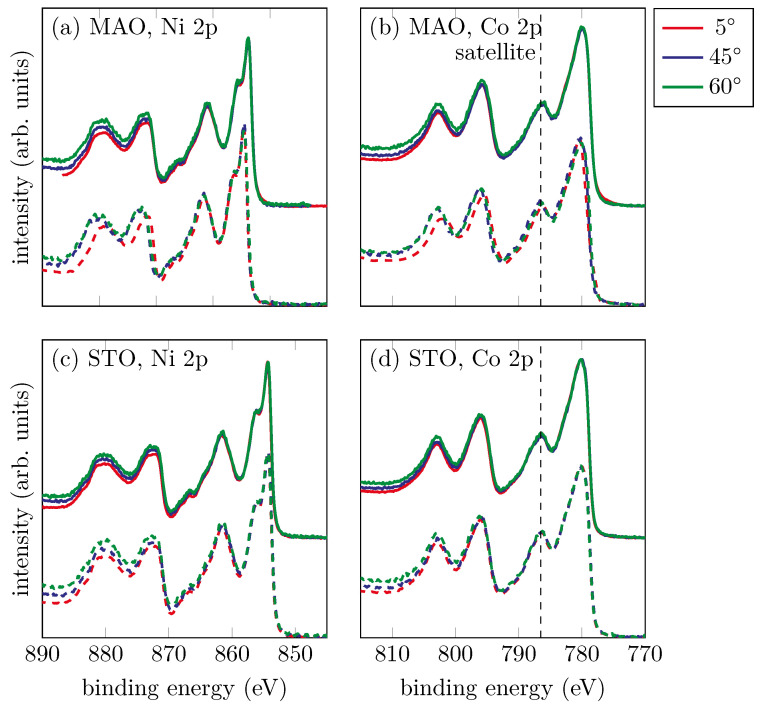
HAXPES measurements for the films on MAO (**a**,**b**) and STO (**c**,**d**) substrates for several photoemission angles. The solid lines represent the films prepared with O_2_ while the dashed lines represent the films prepared with O*. All data were scaled to maximum intensity for better comparability. Regardless of the oxygen used, the shape of the spectra was identical for all films.

To get more information about the stoichiometry and chemical properties in bulk, HAXPES measurements were performed at different photoemission angles. Figure 8 shows sample HAXPES data for the films on the MAO and STO substrates for photoemission angles of 5°, 45° and 60°.

It was quite obvious that all the spectra had an identical shape. Only the background increased for higher photoemission angles, due to lower intensity. Compared with the soft-XPS measurements for films not exposed to ambient conditions, the satellite of the Co 2p${}_{3/2}$ peak (cf. Figure 8 dashed lines) was very distinctive and showed no difference in the two preparation methods. As explained before, the pronounced intensity of this satellite pointed to the formation of CoO. Therefore, we could assume that the formation of NCO with a spinel structure was only stable under vacuum conditions, and exposure to ambient conditions reduced the film. By obtaining different depths of information in HAXPES, it was possible to conclude further that the Ni and Co cations were homogeneously distributed throughout the entire film.

## 4. Conclusions

We prepared stoichiometric NCO films on MgO(001), MAO(001) and STO(001) substrates by means of RMBE and OPA-MBE. In situ soft-XPS and LEED reveal high quality thin films. For the films prepared with oxygen plasma, we found a spinel surface structure in the LEED as well as low intensity for the Co 2p${}_{3/2}$ satellite in soft-XPS, which also indicated an NCO spinel structure. On the other hand, it was not possible to create NCO with a near-surface inverse spinel structure using RMBE. In this case, a NiCo oxide film with an apparent rock salt structure was formed. Thus, the high reactivity of atomic oxygen seemed to be necessary to obtain NCO films.

XRR measurements showed a comparable thickness for all prepared films with the same evaporation parameters. Using O* during evaporation led to a smaller film thickness. Moreover, the RMS roughness was in good agreement with the FWHM of the NCO film reflexes, yet both values were the highest for the MAO substrate although the lattice mismatch was the lowest there.

XRD measurements after exposure to ambient conditions were performed in a θ–2θ geometry. We observed clear Laue fringes for films on the MgO substrate, indicating a highly crystalline ordering and a homogeneous film thickness. These Laue fringes were less distinguishable for the STO substrate and completely vanished for films on the MAO substrate. Therefore, the ordering of the oxide films depended highly on the substrate. The theoretical lattice mismatch of NCO on the various substrates was the lowest for MAO (0.4–1.2%), so it is surprising that the ordering was the worst here and the best for the MgO substrate with a lattice mismatch of −3.7 to −2.87%. After evaluating the oxide Bragg film peak positions we determined the vertical layer distances of the films, which was in good accordance with the pseudomorphic growth of CoNi oxide films with rock salt structure. Therefore, the best lattice matching was obtained for growth on MgO(001). The vertical crystallite grain sizes were calculated from the FWHM of the Bragg positions of the oxide films. The crystallite size was the most pronounced for the MgO substrate and the least for the MAO substrate. To evaluate the formation of the spinel structure of the oxide films, XRD measurements along the (11L) CTR of STO were performed. Whereas film peaks were visible close to the STO(111) and STO(113) Bragg peaks, no peak was visible close to the STO(112) Bragg peak, indicating the formation of a rock salt structure and thus no spinel structure. Additional HAXPES measurements confirmed the previous observations. The satellites of the Co 2p${}_{3/2}$ peak were very distinctive compared to the soft-XPS measurements, therefore pointing to an absence of Co^3+^ cations, which are a characteristic of stoichiometric NCO.

Thus, the preparation of stoichiometric NiCo_2_O_4_ with an inverse spinel structure was successful using a plasma source that formed oxygen radicals. When the films were prepared with molecular oxygen, a rock salt structure was seen in the LEED. However, after exposure to ambient conditions, the film is reduced with contracted crystalline structure and a rock salt structure was observed in all films. A possible solution to this effect could be to prepare a capping layer to protect the surface from external influences.

## Data Availability

The data presented in this study are available on reasonable request from the corresponding author.
